# Source apportionment of circum-Arctic atmospheric black carbon from isotopes and modeling

**DOI:** 10.1126/sciadv.aau8052

**Published:** 2019-02-13

**Authors:** P. Winiger, T. E. Barrett, R. J. Sheesley, L. Huang, S. Sharma, L. A. Barrie, K. E. Yttri, N. Evangeliou, S. Eckhardt, A. Stohl, Z. Klimont, C. Heyes, I. P. Semiletov, O. V. Dudarev, A. Charkin, N. Shakhova, H. Holmstrand, A. Andersson, Ö. Gustafsson

**Affiliations:** 1 ACES—Department of Applied Environmental Science and the Bolin Centre for Climate Research, Stockholm University, Svante Arrhenius Väg 8, 10691 Stockholm, Sweden.; 2 The Institute of Ecological, Earth, and Environmental Sciences, Baylor University, Waco, TX, USA.; 3 Department of Environmental Science, Baylor University, Waco, TX, USA.; 4 Climate Research Division, Atmospheric Science and Technology Directorate, Environment and Climate Change Canada, 4905 Dufferin Street, Toronto, ON M3H 5T4, Canada.; 5 Department of Geosciences and the Bolin Centre for Climate Research, Stockholm University, Svante Arrhenius Väg 8, 10691 Stockholm, Sweden.; 6 NILU—Norwegian Institute for Air Research, Instituttveien 18, 2027 Kjeller, Norway.; 7 IIASA—International Institute for Applied Systems Analysis, Schlossplatz 1, 2361 Laxenburg, Austria.; 8 Pacific Oceanological Institute, Russian Academy of Sciences, 43 Baltiyskaya Street, 690041 Vladivostok, Russia.; 9 International Arctic Research Center, University of Alaska Fairbanks, 930 Koyukuk Drive, Fairbanks, AK, USA.; 10 Tomsk National Research Polytechnic University, 43 A Lenina Ave., 634034 Tomsk, Russia.

## Abstract

Black carbon (BC) contributes to Arctic climate warming, yet source attributions are inaccurate due to lacking observational constraints and uncertainties in emission inventories. Year-round, isotope-constrained observations reveal strong seasonal variations in BC sources with a consistent and synchronous pattern at all Arctic sites. These sources were dominated by emissions from fossil fuel combustion in the winter and by biomass burning in the summer. The annual mean source of BC to the circum-Arctic was 39 ± 10% from biomass burning. Comparison of transport-model predictions with the observations showed good agreement for BC concentrations, with larger discrepancies for (fossil/biomass burning) sources. The accuracy of simulated BC concentration, but not of origin, points to misallocations of emissions in the emission inventories. The consistency in seasonal source contributions of BC throughout the Arctic provides strong justification for targeted emission reductions to limit the impact of BC on climate warming in the Arctic and beyond.

## Teaser

Isotopes pinpoint strong seasonal variations in black carbon sources with consistent patterns at sites around the Arctic.

## INTRODUCTION

Black carbon (BC) aerosols, originating from incomplete combustion of fossil fuels and biomass, contribute to the increased rates of warming of the Arctic (*1*–*3*). Policy-focused research suggests that collaboration and alliances of even small groups of countries could achieve urgently needed, efficient, rapid, and substantial BC mitigation (*4*). Atmospheric transport models—fundamental for validation of inventories used in climate policy discussions—have difficulties in accurately reproducing Arctic BC concentrations (*5*–*7*). Comparison of model predictions with source-diagnostic observations offers a means to better understand the emissions of BC reaching the Arctic (*8*–*10*). Source attributions are challenged both by a lack of observational constraints and by large uncertainties in emission inventories, the latter being a key element for modeling transport and climate effects of BC, specifically in the Arctic (*8*, *11*, *12*).

**Fig. 1:**
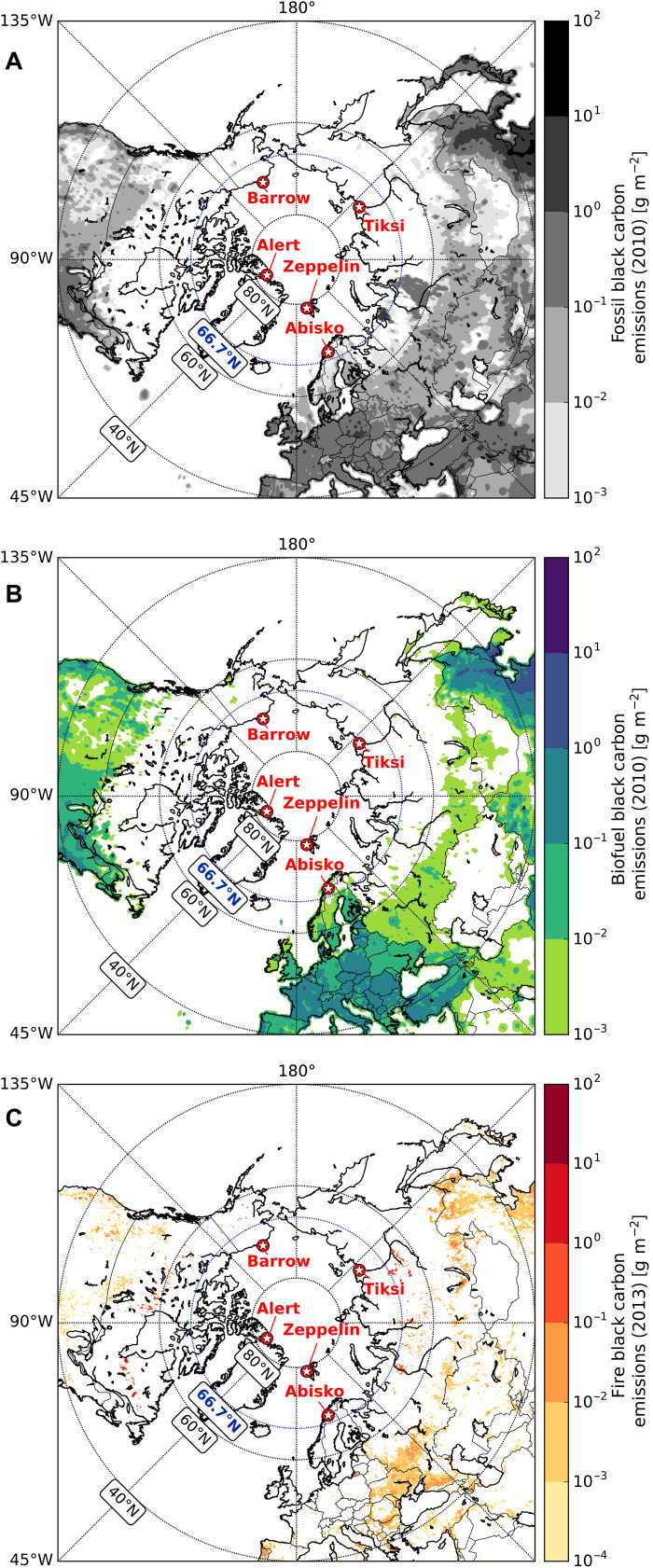
Annual BC emissions and all circum-Arctic sites from emission inventories. The five Arctic stations are marked in red: Abisko (Sweden), Alert (Canada), Barrow (United States), Tiksi (Russia), and Zeppelin (Norway). Emission data in the maps are log scale. (**A**) Fossil fuel BC emissions (ECLIPSEv5 base year 2010). (**B**) Biofuel BC emissions (ECLIPSEv5 base year 2010). (**C**) BC emissions from open fires (GFED4.1s data for observational year 2013).

Observation-based Arctic BC studies are scarce and rarely extend over more than 1 year (*13*–*15*), especially with regard to data on source-diagnostic dual-isotopic composition (δ^13^C and Δ^14^C). Hereafter, BC is used when referring to model results or the aerosol in general, and elemental carbon [EC; the mass-based BC analog (*16*)] is used when referring specifically to observational data. The present study provides new year-round δ^13^C/Δ^14^C-based source apportionment of EC from the Arctic sites Alert (Canadian High Arctic; *n* = 9), Zeppelin (Svalbard; *n* = 11), and Barrow (north Alaska; *n* = 10), covering a period of ~3 years. To provide a comprehensive circum-Arctic perspective (Fig. 1), these three records are combined with our recently published studies of EC aerosol concentrations and isotopic signatures from two long-term campaigns from Abisko (northern Scandinavia; *n* = 17) (*7*) and Tiksi (northeast Siberia; *n* = 17) (*17*) and a winter study (*n* = 6) from Barrow (*18*). The ^14^C/^12^C isotope ratio of an EC sample allows determination of the biomass burning fraction (*f*_bb_; containing contemporary ^14^C) relative to the fossil fuel combustion fraction (*f*_fossil;_ devoid in ^14^C) (*19*). The ^13^C/^12^C ratio helps to further distinguish between various types of fossil fuel sources [e.g., natural gas, coal, or oil (*17*)]. Last, these observations of atmospheric BC are compared with results from an atmospheric transport model, which includes both anthropogenic and natural-fire BC emissions, and has shown great potential to accurately simulate observational data (*5*, *7*).

## RESULTS

### BC concentrations

**Fig. 2:**
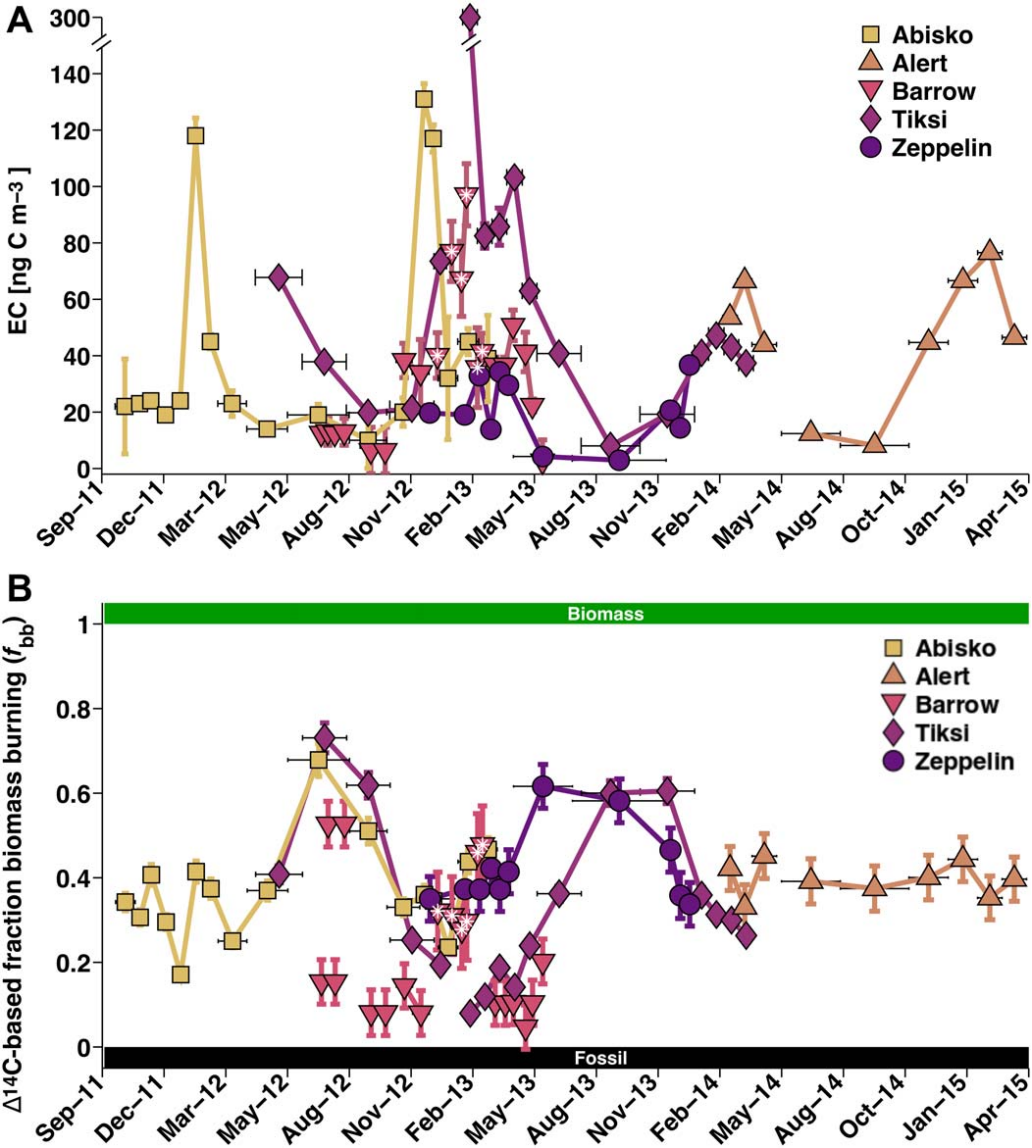
Circum-Arctic EC observations between 2011 and 2015. From light to dark: Abisko (squares), Alert (upward triangles), Barrow (downward triangles), Tiksi (diamonds), and Zeppelin (circles). Horizontal bars (black) indicate sampling duration. Vertical error bars show observational uncertainties (1 SD). Data from Barret *et al*. (*18*) are marked with white asterisks. (**A**) EC concentrations. Notice that one high-EC sample from Tiksi (~300 ng C m^−3^) is off-chart. (**B**) EC source apportionment expressed as fraction of biomass burning of EC.

All sites displayed a seasonal pattern of low EC concentrations in summer (July to September) and higher concentrations during the rest of the year, peaking in the winter/spring “Arctic haze” (*20*) period (Fig. 2). Annual averages (Table 1) were relatively uniform and resulted in an EC value for the circum-Arctic of 28 ± 24 ng C m^−3^. The observed differences in EC concentrations from one site to the other occur because of different proximity to EC sources, site specificity for various types of carbonaceous sources, and differences in aerosol lifetime. The latter is affected by many factors, including differences in size and mixing state of primary aerosols, air mass transport pathways, wet and dry deposition during transport, and orography of the terrain (*21*). However, some shorter periods were observed where some of the stations had very similar EC concentrations (table S1). In winter, when BC emissions are increased, differences in BC sources between sites are more pronounced, removal processes are least effective, and transport patterns differ from summer conditions (*22*); all these factors combine to generate larger local differences in Arctic haze concentration in winter months.

**Table 1:** Annual observational data (selected period) for the Arctic observatories. EC concentrations and SDs are volume-weighted, whereas the fraction that is biomass burning (*f*_bb_) and δ^13^C values (and their SDs) are mass-weighted. For the Arctic mean, a value is given without Barrow due to the shorter (less than one full year) period of the data coverage (table S5).

**Site**	**(DD/MM/YY)**	**Days**	**EC (ng/m^3^)**	***f*_bb_ (−)**	**δ^13^C (‰)**
Alert	05/03/14 to 18/03/2015	371	36 ± 28	0.40 ± 0.05	−27.9 ± 0.8
Abisko	20/12/11 to 19/12/12	363	27 ± 32	0.42 ± 0.14	−26.3 ± 1.4
Barrow	16/07/12 to 04/06/13 (not continuous)	224	25 ± 20	0.15 ± 0.13	−27.5 ± 1.5
Tiksi	27/02/13 to 07/03/14	373	38 ± 29	0.30 ± 0.17	−28.4 ± 0.8
Zeppelin	15/11/12 to 22/11/13	370	12 ± 11	0.41 ± 0.09	—
Arctic	All above		28 ± 24	0.29 ± 0.16	−26.8 ± 1.6
Arctic	Without Barrow		31 ± 27	0.39 ± 0.10	−27.0 ± 1.2

### Fossil fuel and biomass burning sources from radiocarbon

All stations exhibited clear seasonality in BC sources, with a dominant contribution from fossil fuel–based emissions (Fig. 2). The annual mean radiocarbon-based “fraction biomass burning” (*f*_bb_ = 1 – “fraction fossil fuel”) of EC for the circum-Arctic was 39 ± 10%, excluding Barrow, for which there exists less than a full year’s coverage, and 29 ± 16% including Barrow. The *f*_bb_ values for the single sites ranged from 15 to 42% (Table 1). Variations are best described in seasonal patterns (table S1). Northward transport of pollutants carried predominantly fossil fuel emissions in the polluted winter (December-January-February; *f*_bb_ of 10 to 40%), whereas biomass burning sources were relatively more important during the pristine summer months (June-July-August; 33 to 68%) (*23*). The mean contribution of biomass burning to the Arctic EC was 25 ± 16% in the polluted winter and 42 ± 19% in the much cleaner summer (table S1). Barrow showed the same seasonal variation in *f*_bb_ as Abisko, Tiksi, and Zeppelin sites, but the fossil fuel fraction of BC was generally higher_._ The surface boundary layer in Barrow is usually isolated from wildfires in central Alaska by the Brooks Range, separating the leeward tundra and wetland of the North Slope from the rest of boreal Alaska. Biomass burning plumes are lofted at the Brooks Range and do not always descend again to the surface within the North Slope. Most of the stations had relatively long periods of overlapping data, except for Alert. There, only the first two samples (i.e., composites) overlap with the final two Tiksi composites while having almost identical sampling times. In terms of *f*_bb_, this bridging period shows a consistent transition from the preceding Tiksi into Alert observations. However, the succeeding *f*_bb_ seasonality at Alert (40 ± 5%) is much weaker and oscillates around the annual Arctic mean *f*_bb_ determined in this study (Table 1). A possible explanation for Alert’s weak seasonality could be its location, which is furthest from BC sources among the Arctic stations. The low annual variation in accumulation mode particles (*24*) at the most remote site, Alert, suggests that arriving aerosols are more mixed during their longer transport compared to the other sites, creating a relatively constant *f*_bb_ signal.

### Combustion sources apportioned by stable carbon isotopes

**Fig. 3:**
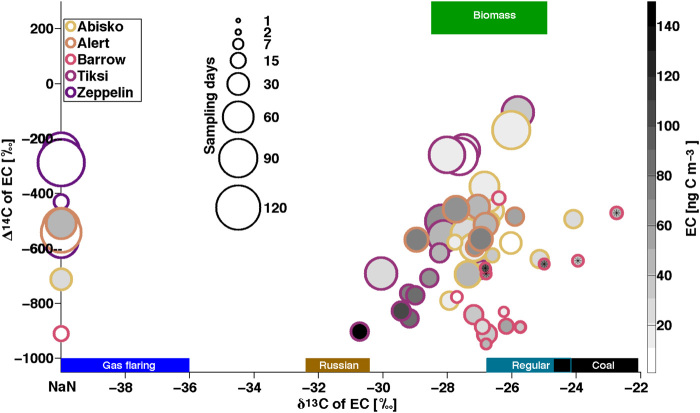
Multidimensional source apportionment plot of the Δ^14^C versus δ^13^C isotopic signature of samples from all stations. The colored squares show the endmember ranges for the different EC sources of biomass burning (green) and the fossil fuels: gas flaring (blue), liquid fossil fuels of Russian origin (brown), liquid fossil fuels of “regular” (defined as American, Chinese, and western European emission) origin (dark cyan), and coal (black). Data from Barret *et al*. (*18*) are marked with a black asterisk. From light to dark coloring: Abisko, Alert, Barrow, Tiksi, and Zeppelin. The degree of shading in the circles indicates the EC concentration for each sample (right shading bar). The areas of the circles indicate the sampling time in days from 1 to 120. Samples for which no δ^13^C data were available (e.g., all Zeppelin data) are placed on the *y* axis. Uncertainties and error bars are not shown (can be received from tables in the Supplementary Materials) and are smaller than the diameter of the circles.

In addition to radiocarbon data, the stable carbon isotopic ratio (δ^13^C) provides additional insight into source apportionment, especially between different fossil fuel source classes (i.e., coal versus liquid fossil fuel versus gas flaring). Liquid fossil fuel sources can be further deconvoluted with δ^13^C-EC fingerprinting. EC emissions of Russian origin are more depleted in ^13^C compared to emissions of Chinese and western European (“regular”) liquid fossil fuels (*17*, *25*). The most δ^13^C-depleted annual signature of EC was found in Tiksi, followed by Alert, Barrow, and Abisko (Table 1; no δ^13^C data are available for Zeppelin due to low carbon content). Alert and Tiksi had the narrowest δ^13^C SD (both ±0.8‰; Fig. 3), with a wider SD for Abisko and Barrow (roughly ±1.5‰). Taking into account the uncertainty of the potential sources, distributions smaller than 1‰ can be considered narrow, as the “pure” fossil endmembers (e.g., coal) have an uncertainty range of 1 to 1.8‰; the endmember range for gas flaring is more uncertain (±3‰) (*17*). Although narrow ranges in annual δ^13^C point to well-mixed air masses arriving at a site, influence from local point sources cannot be excluded. Point-source signals are difficult to detect, because of the long consecutive sampling times that had to be used in this study to allow collection of enough carbon material to enable radiocarbon analysis. For the 2 years of Tiksi observations, only spring 2013 showed a clear influence from local liquid fossil fuel emissions of Russian origin (*17*). This finding is essential, because significant fossil fuel emissions appear to be absent in the emission inventory within a large radius from Tiksi (Fig. 1A). In contrast to the radiocarbon data, no distinct seasonality for stable isotopic EC signatures was observed for the circum-Arctic. Gas flaring, which is mostly of Russian origin (*12*, *26*, *27*) and was suggested earlier to be a major source of the Arctic BC surface concentration (*26*), did not appear to be abundant during the observed periods and under the given limitations of the current gas-flaring isotopic endmember (text S1) (*17*).

### Model performance

**Fig. 4:**
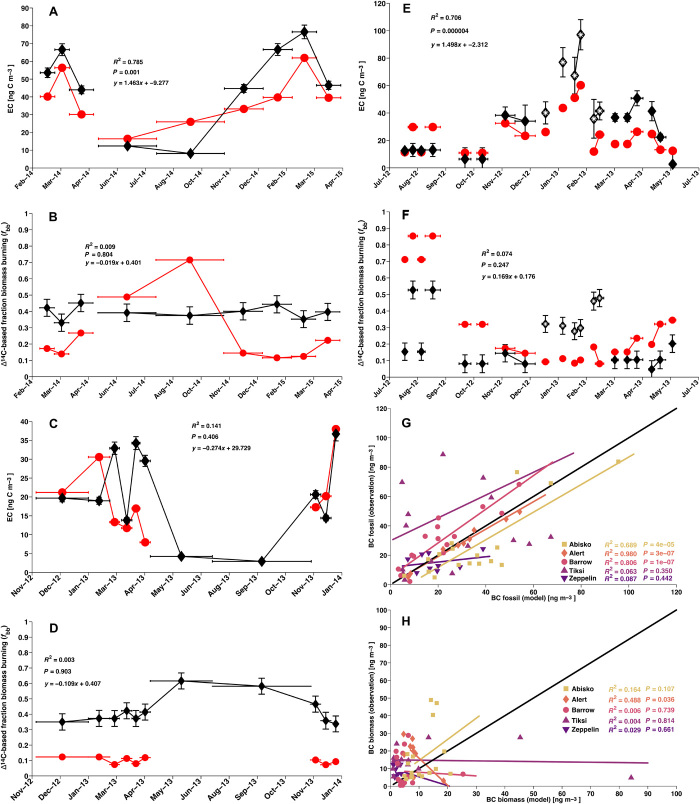
Alert, Barrow, Zeppelin, and BC of fossil versus BC of biomass burning. (**A** to **F**) Observation (black line) and model (red line). EC is used synonymous to BC. Horizontal bars indicate sampling duration. Vertical error bars show observational uncertainties (1 SD). (A) Alert BC concentrations. (B) Alert fraction biomass burning. (C) Zeppelin BC concentrations. (D) Zeppelin fraction biomass burning. (E) Barrow BC concentrations. Data from Barret *et al*. (*18*) are marked with a white asterisk. (F) Barrow fraction biomass burning. Data from Barret *et al*. (*18*) are marked with a white asterisk. The linear fit (*R*^2^) and *P* value between model and observation are shown in each respective panel. (**G** and **H**) From light to dark: Abisko (squares), Alert (diamonds), Barrow (circles), Tiksi (upward triangles), and Zeppelin (downward triangles). The linear fits (*R*^2^) and *P* values between mode and observation are shown for all stations in each respective panel. (G) BC fossil (fuel) concentrations are the product of 1 − *f*_bb_ and BC concentration. (H) BC biomass (burning) is the product of *f*_bb_ times BC concentration.

**Fig. 5:**
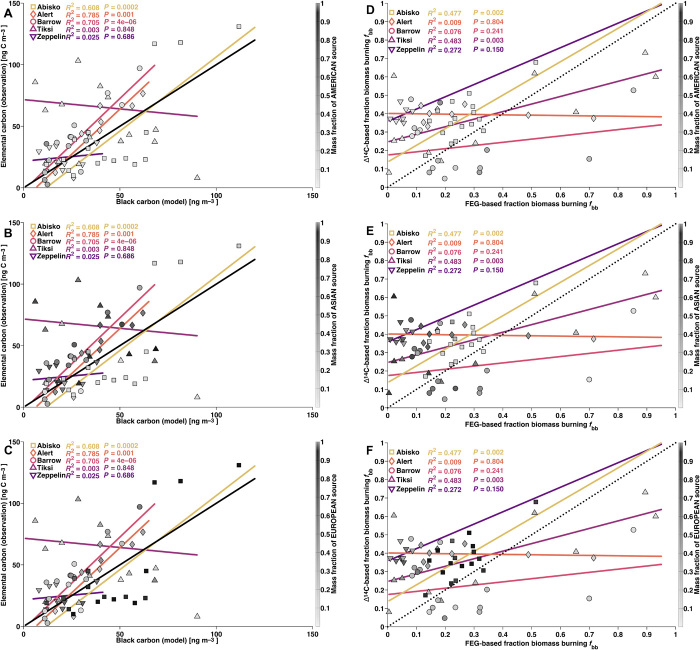
Model versus observation. From light to dark: Abisko (squares), Alert (diamonds), Barrow (circles), Tiksi (upward triangles), and Zeppelin (downward triangles). The linear fits (*R*^2^) and *P* values for EC and BC are replicated in each panel. The color bar (and gray shaded symbols) represents the fraction of simulated anthropogenic sources (fossil fuel and biofuel) by continent, separated in the three panels. (**A**) Mass fraction of simulated BC from (North) American sources. (**B**) Mass fraction of simulated BC from Asian sources. (**C**) Mass fraction of simulated BC from European sources. (**D** to **F**) Biomass burning fraction of EC, based on radiocarbon measurements versus biomass burning fraction based on FEG model simulations. The linear fits (*R*^2^) and *P* values for fraction biomass burning from observation (Δ^14^C) and model (FEG) are replicated in each panel. The color bar (and gray shaded symbols) represents the fraction of simulated anthropogenic sources (fossil fuel and biofuel) by continent, separated in the three panels. (D) Mass fraction of simulated BC from (North) American sources. (E) Mass fraction of simulated BC from Asian sources. (F) Mass fraction of simulated BC from European sources.

A Lagrangian atmospheric transport model [FLEXible PARTicle dispersion model (FLEXPART) (*28*)], based on an anthropogenic emission inventory [Evaluating the Climate and Air Quality Impacts of Short-Lived Pollutants (ECLIPSE) (*12*)] and an open fire emissions inventory [Global Fire Emissions Database (GFED) (*29*)], was used to simulate BC concentrations at the five measurement sites, enabling the investigation of BC’s geographical origins (fig. S1). Furthermore, simulated BC concentrations were split into anthropogenic (biofuel or fossil fuel) and natural (open fires and wildfires) contributions. There were observation model offsets, with both over- and underestimation in model predictions relative to the observations—both in BC concentrations and especially in the BC source apportionment (Fig. 4, A to F) (*7*, *17*). The model performed better for fossil fuel–BC concentrations than for biomass burning–BC (Fig. 4, G and H). Better performance was achieved for sites (Abisko, Alert, and Barrow) with fully overlapping model and observational data coverage, and where no clear influence of local emissions was observed. The influence of missing local emissions and relatively large discrepancies for BC sources (i.e., fossil fuel versus biomass burning; *f*_bb_), but otherwise well-simulated BC concentrations, suggests large uncertainties for geographical allocation (i.e., misallocation) and composition of BC sources in the emission inventories. In addition to uncertain emissions, the transport of BC into the deep Arctic is a difficult process to model because it involves complex interactions of dry deposition and precipitation scavenging as well as diabatic transport in the low sunlit Arctic with a strong surface-based inversion (*5*). The difference between Barrow and other Arctic sites also indicates the importance of accurately modeling orographic impacts on surface concentrations. If global transport models with larger grid sizes (2.5° × 2.5°) are used to simulate regional transport over complex terrain like the Brooks Range, the model will be biased high unless nested grids are incorporated to capture dilution during uplift (*30*). For example, a recent global-scale GEOS-Chem modeling study suggested high biomass burning contributions for Barrow in the summer; however, parallel measurements did not reflect a similarly large increase in surface BC concentrations (*27*). There may also be local influence from nearby Utqiaġvik. However, the model skill was influenced by the projected geographical origin of BC, showing better agreement for European sources (Fig. 5).

### Geographical BC sources

Two available outputs from FLEXPART are the “potential emission sensitivity” function and a “source contribution function.” The latter can be obtained when FLEXPART is coupled to an emission inventory, such as ECLIPSE. The potential emission sensitivity is proportional to particles’ residence time, in units of seconds per volume (or area), in a particular model grid cell. Close to the surface (0- to 100-m altitude), the potential emission sensitivity is also called footprint emission sensitivity (*31*). The source contribution function, with units of mass per second, is a measure for the quantity of how much a source in an emission inventory grid cell would contribute to the total concentration at a receptor site. That way, both the geographical sources and the measured concentrations—the product of potential emission sensitivity and source contribution function—can be simulated for an observational site. Reversely, a latitudinal cutoff can be calculated, pointing to a fraction [e.g., 90% (*4*)] of BC emissions that come north or south of that boundary. The FLEXPART model indicates that main source regions for 90% of the simulated annual anthropogenic (non-open biomass burning) BC in the circum-Arctic were north of 42° latitude. This includes all Arctic council members (Canada, Denmark, Finland, Iceland, Norway, Russia, Sweden, and the United States), most of Europe, some post-Soviet states (Belarus, Kazakhstan, Moldova, and Ukraine), and northern China. The remaining ~10% BC south of 42°N originated mostly from China. Together, for the selected annual periods and stations, the modeled total BC from anthropogenic and natural sources were 38 ± 25% European (including western Russia), 36 ± 25% Asian, and 3 ± 11% North American, with the remainder (23 ± 24%) coming from global open biomass burning (table S2). Wildfires that affect the Arctic occur to a big part in Asia (*32*). Barrow was the only receptor site where the transport model projected significant impacts from anthropogenic American sources (17 ± 11%). The influences from Asian emissions were relatively high at Alert (50 ± 16%), Barrow (43 ± 21%), Tiksi (41 ± 25%), and Zeppelin (41 ± 12%), whereas European emissions dominated at Abisko (84 ± 6%) and Zeppelin (57 ± 14%) and were pronounced at Alert (27 ± 10%). By assigning these source contributions, it is also worth noting that the countries and regions mostly responsible for emissions (China, Europe, Russia, and the United States) would also benefit most from mitigation efforts in the short term, with regard to socioeconomic impact (health benefits and avoided crop loss) (*4*).

## DISCUSSION

Overall, the model simulations agreed fairly well with this extensive set of observed circum-Arctic concentrations, thus providing important observational support for model-based mitigation plans. In contrast, however, the model-derived source apportionments (e.g., expressed as *f*_bb_) were in less agreement with observed *f*_bb_. This discrepancy is likely due to (i) misallocation of anthropogenic BC sources within the Asian regions, (ii) missing anthropogenic sources (*12*, *17*, *33*), (iii) uncertainties in estimates of BC emissions from wildfires (*32*), and (iv) uncertainties in atmospheric transport modeling. However, there is good agreement between the model and observations when comparing fossil-only BC concentrations (Fig. 4G), at least for the sites where there are no missing meteorological data [due to the increase of vertical model resolution of the European Centre for Medium-Range Weather Forecasts (ECMWF) in 2013] or absent local emissions in the inventory. This suggests that improved estimates of biomass emissions, including wildfires and domestic activities, are an important factor for further constraining the impact of BC on Arctic climate. The observational data show that both fossil and biomass emissions contribute substantially to the levels of BC in the Arctic, but with opposite seasonal trends. This is reliable and essential information for informed policy decisions toward targeted emission reductions, and in support of collaboration and alliance between small clubs of countries.

## MATERIALS AND METHODS

### Experimental design

A homebuilt high-volume aerosol sampler was used at the Dr. Neil Trivett Global Atmosphere Watch Observatory in Alert, Canada [83.2°N, 62.5°W, 210 m above sea level (masl)] to collect 44 samples between 4 February 2014 and 15 April 2015. The sampler was installed at a walk-up deck, about 4 m above the ground. Flow rate was approximately 1.4 m^3^ min^−1^ at standard temperature and pressure condition. Quartz filters (8 in × 10 in; QFF, Millipore, USA) were sampled continuously with sampling times of 7 days from December to April and 14 days from May to November (with one interruption from 30 April 2014 to 5 May 2014). A total of 10 field blanks (roughly one every month) were collected. After sampling, filters were stored (at room temperature ~20°C) in their sampling cartridges (inside sealed plastic bags) at the Alert station and shipped in aluminum boxes (containing five sampling cartridges each) to Stockholm, where they were transferred into precombusted Al foil and stored at −20°C. Air temperatures and pressures have been recorded and averaged over the integrated sampling time, and both were used for final flow rate and total air volume calculation.

A yearlong sampling campaign was conducted at the Department of Energy Atmospheric Radiation Measurement Climate Research Facility, 7.4 km northeast of the village of Utqiaġvik (formerly known as Barrow), AK (71.3°N, 156.6°W, 11 masl), on the North Slope of Alaska from 16 July 2012 to 4 June 2013. Samples were collected continuously with no exclusion based on local wind direction. The campaign was designed to monitor local and regional influences on ambient concentration and physical properties (i.e., sources from the village of Utqiaġvik were not excluded from the study). Particulate matter with an equivalent aerodynamic diameter less than 10 μm (PM_10_) was collected on precombusted QFF (20 cm × 25 cm, Tissuquartz Filters 2500 QAT-UP) using a Tisch high-volume PM_10_ sampler (TE-6070, Tisch Environmental, Cleves, OH). All samples were stored in aluminum foil packets and Ziploc storage bags in a freezer (−10°C) before and after sampling. Filter blanks were collected at least once a month or when sampler maintenance was conducted. All blanks (*n* = 15) were handled in the same manner as samples.

High-volume aerosol samples were collected on the roof of the Zeppelin Observatory, Svalbard, Norway (78.9°N, 11.9°E, 478 masl). Aerosol samples (PM_10_) were collected on QFF (8 in × 10 in; Millipore, USA) from 16 June 2012 to 30 December 2013, with two 3-day interruptions. A total of 33 filter samples and 11 blanks were collected. Early summer aerosol concentrations were very low; hence, for isotope analysis, a subset from 15 November 2012 to 30 December 2013 (410 days) was selected (table S7). Filters were kept in precombusted Al foil and stored at −20°C.

This temporary Abisko receptor site was located 10 km east of the village and research station of Abisko in northern Sweden (68.4°N, 19.1°E, 359 masl), as previously described (*7*). Briefly, samples were collected from 29 September 2011 to 27 March 2013 on precombusted QFF filters (8 in × 10 in; Millipore, USA) using a PM_2.5_ inlet high-volume sampler (model DH77, Digitel AG) with filter-changing intervals of 12 to 28 days, depending on the season and weather conditions.

The Tiksi sampling site, Polar Geocosmophysical Observatory (71.4°N, 128.5°E, 35 masl), is situated ~10 km southwest of the Tiksi settlement, has been in operation since 1958, and is run by permanent technical staff from the Russian Academy of Sciences, as previously described (*17*). Aerosol sampling of total suspended particles on precombusted QFF filters (8 in × 10 in; Millipore, USA) was performed continuously for ~24 months (16 April 2012 to 7 March 2014) with sample intervals of 15 to 25 days, depending on the weather conditions.

### EC analysis

The EC and organic carbon (OC) concentrations were determined by a thermal-optical transmission (TOT) analyzer (instrument #227, Sunset Laboratory, Tigard, OR) using the National Institute for Occupational Safety and Health 5040 protocol (*34*). This method is known to potentially overcorrect charring fraction relative to other methods and to underestimate the EC content (*35*–*39*). During charring, parts of the OC could also end up in the EC fraction in the form of pyrogenic carbon, which would then influence the isotopic composition of the EC fraction. This effect was evaluated by a sensitivity analysis in a previous study, which concluded that the radiocarbon-derived fraction of biomass burning could be overestimated by a maximum of 7%, in extreme cases (*25*). The detection limit for EC was based on the OC concentration of the field blanks, which had ~100 ng C cm^−2^ OC (this roughly translates to 2 ng C m^−3^ for Zeppelin samples, which had the lowest EC concentrations measured). EC could not be detected in any of the blanks.

Annual mean concentrations were calculated as

$$\bar{\text{EC}}=\frac{\sum_{i=1}^{n}\text{EC}(i)\cdot V(i)}{\sum_{i=1}^{n}V(i)}$$ (1)

where EC is the EC concentration unit (ng C m^−3^), *V* is the volume collected for the respective aerosol filter sample, and *i* is the sample index.

### Carbon isotope analysis

Before analysis, the filter samples were acid-fumigated with 12 M HCl (inside a desiccator for 24 hours and subsequently dried at 60°C for 1 hour) to remove carbonates and to prevent their charring effect during pyrolysis (*19*, *25*). The EC fraction, generated through the TOT analyzer, was isolated after CO_2_ conversion and cryogenically trapped using a modified Sunset Laboratory instrument (*40*). Offline analysis of the carbon isotopes was conducted using accelerator mass spectrometry (AMS) at the U.S. National Science Foundation National Ocean Science Accelerator Mass Spectrometry (NOSAMS) facility (Woods Hole, MA) (*41*, *42*). The six Barrow winter samples from Barrett *et al*. (*18*) were prepped for radiocarbon analysis using a previous EC isolation method. In this method, the TOT analyzer parameters were truncated to preserve EC on the filter samples rather than being combusted to CO_2_. This preserved EC was then sent to NOSAMS, where it was combusted to CO_2_ and analyzed using the AMS method mentioned above.

The relative contributions of EC to biomass burning *f*_bb_ and fossil fuel combustion (*f*_fossil_ = 1 − *f*_bb_) were calculated using an isotopic mass balance equation (*19*)

$$\Delta^{14}C=\Delta^{14}C_{\text{bb}}f_{\text{bb}}+\Delta^{14}C_{fossil}(1-f_{\text{bb}})$$ (2)

where Δ^14^C represents the radiocarbon signature in the sample, Δ^14^C_bb_ is the endmember of the contemporary radiocarbon, and Δ^14^C_fossil_ is −1000‰ by definition, as fossil carbon is completely devoid of ^14^C. The contemporary radiocarbon signature (Δ^14^C_bb_) depends on the biomass type, age, and year of harvest. Current monthly mean Δ^14^CO_2_ signatures are below 30‰ (*43*). In case of the Eurasian Arctic stations (Abisko, Tiksi, and Zeppelin) and Alert, an endmember of +225 ± 60‰ is suggested, representing typical Northern tree species (*25*), the most common form of biomass burning fuel. Because of the different source origins (North America), the Δ^14^C endmember used to determine contemporary carbon contributions for the Barrow station was +107.5 ± 50‰ based on wood burning for temperate regions in 2010 (*18*, *44*). These conservative estimates for endmember uncertainty took count of biomass burning sources other than wood [e.g., agricultural waste burning (AWB)] and introduced an additional *f*_bb_ variability of <5%.

Yearly annual mean of the fraction of biomass burning (EC mass-weighted *f*_bb_) was calculated as

$$\bar{f_{\text{bb}}}=\frac{\sum_{i=1}^{n}f_{\text{bb}}(i)\cdot \text{EC}(i)\cdot V(i)}{\sum_{i=1}^{n}\text{EC}(i)\cdot V(i)}$$ (3)

where EC is the EC concentration, *V* the volume collected for the respective sample, and *i* is the sample index.

### Pooling of samples for isotopic analysis

Pooling was a necessity to enable year-round radiocarbon analysis, in terms of the collection of enough carbon material, complete the laboratory work in a reasonable time, and stay within a reasonable budget (due to rather costly AMS analysis). A subset of 43 Alert samples (12 February 2014 and 15 April 2015) was pooled into nine composites, considering season and previously analyzed composites at Tiksi (to get two data points of identical start and stop dates). Samples were pooled to achieve 40 to 80 μg of EC, with volume normalized mass from each sample.

Multiple PM_10_ samples from Barrow were pooled for radiocarbon analysis. This pooling was accomplished considering season and source region. Samples from the same seasons and source regions (Russian Arctic, Canadian Arctic, Arctic Ocean, and interior Alaska) were composited (based on HYSPLIT back-trajectory analysis). Samples were pooled to achieve 60 μg of EC, with volume normalized mass from each sample.

The 24 Zeppelin filters collected during 15 November 2012 and 30 December 2013 were pooled into 11 composites (*7*). None of the stable carbon data could be obtained; hence, only Δ^14^C data were reported. Samples were pooled to achieve 40 μg of EC, with volume normalized mass from each sample.

All Abisko samples were pooled into 17 composites/samples. Higher temporal resolution was chosen during Arctic Haze seasons (winter/spring), and lower resolution was chosen for the summer months, as previously described (*7*). The 17 Tiksi composites were pooled with emphasis on higher temporal resolution during the Arctic haze period, as previously described (*17*).

### FLEXPART-ECLIPSE-GFED model

For the bottom-up estimates of the BC concentrations, the FLEXPART-ECLIPSE-GFED (FEG) model was used, consisting of the atmospheric dispersion model FLEXPART (*28*, *45*), coupled to the ECLIPSE (*12*) emission inventory and satellite-based open fire emissions by GFED (*29*). FLEXPART version 9.2 was run in backward mode for the same location and time periods over which the measurements were taken. A logarithmic size distribution with mean particulate diameters of 250 nm was used, with a logarithmic SD of 1.25. Simulations extended over 20 days back in time, which is sufficient to include most emissions injected into an air mass arriving at the station, given a typical BC lifetime of roughly 1 week. The simulations used meteorological analysis data from the ECMWF at a resolution of 1° × 1° latitude/longitude. Data in the summer and fall of 2013 were missing due to ECMWF’s increase of vertical model resolution on 25 June 2013. FLEXPART accounts for dry deposition and wet scavenging, differentiating between below-cloud and in-cloud scavenging. Anthropogenic BC emissions were received from the ECLIPSE version 5 emission inventory (*12*), which is based on the GAINS (greenhouse gas–air pollution interactions and synergies) model (*46*). The emissions were available at yearly resolution for the various source types and, in addition, contained an explicit split between biofuel (modern) and fossil fuel emissions.

To estimate the biomass burning contribution from open fires (including wildfires and AWB), the most recent version (4.1s) of GFED was applied (*29*). This satellite-based emission inventory was used with monthly resolution, and the spatial resolution was changed from 0.25° to 0.5° to match ECLIPSE’s resolution. Emissions from wildfires were not accounted for by the ECLIPSE model. However, AWB is included in ECLIPSE as biofuel. Hence, the AWB fraction of ECLIPSE was removed to avoid double counting.

The yearly mean *f*_bb_ for the available FEG data was calculated as

$$\bar{f_{\text{bb}}}=\frac{\sum_{1}^{n}f_{\text{bb}}(i)\cdot t(i)\cdot \text{BC}(i)}{\sum_{1}^{n}t(i)\cdot \text{BC}(i)}$$ (4)

where *f*_bb_ is the model-based fraction of biomass burning BC, BC is the BC concentration, *t* is the sampling time for the respective sample, and *i* is the sample index. The model-derived *f*_bb_ contains all contemporary fuels (i.e., biofuels, wildfires, and AWB).

### Statistical analysis

The total number of samples and composites are mentioned in each respective section and in the Supplementary Materials for all observatories. The coefficients of determination (*R*^2^) used in this work are from linear regression to compare model and observation results. *P* values are also reported in the figures, where applicable, and can be evaluated on a case-by-case basis.

## Supplementary Material

http://advances.sciencemag.org/cgi/content/full/5/2/eaau8052/DC1

Download PDF

Source apportionment of circum-Arctic atmospheric black carbon from isotopes and modeling

## SUPPLEMENTARY MATERIALS

Supplementary material for this article is available at http://advances.sciencemag.org/cgi/content/full/5/2/eaau8052/DC1 Text S1. Gas-flaring uncertainties. Fig. S1. Continental borders considered for the geographical sources in the FEG model. Table S1. Seasonal observational data for the circum-Arctic. Table S2. Simulated fraction of BC mass from global natural (fire) and regional anthropogenic (biofuel and fossil fuel) sources. Table S3. Observational data for Alert. Table S4. Observational data for Abisko. Table S5. Observational data for Barrow. Table S6. Observational data for Tiksi. Table S7. Observational data for Zeppelin. Table S8. Simulated fraction of BC mass (nonweighted) from global natural (fire) and regional anthropogenic (biofuel and fossil fuel) sources. References (*47*–*49*)
