# Supplementary material for: Source apportionment of circum-Arctic atmospheric black carbon from isotopes and modeling
Source: Sci Adv. 2019 Feb 13;5(2):eaau8052. doi: 10.1126/sciadv.aau8052 (PMC6374108; doi:10.1126/sciadv.aau8052)
Supplement: http://advances.sciencemag.org/cgi/content/full/5/2/eaau8052/DC1 [file supp_5_2_eaau8052__index.html]

Science Advances | Science AdvancesAAASSearchScience AdvancesMenu

## Supplementary Materials

**This PDF file includes:**

- Text S1. Gas-flaring uncertainties.
- Fig. S1. Continental borders considered for the geographical sources in the FEG model.
- Table S1. Seasonal observational data for the circum-Arctic.
- Table S2. Simulated fraction of BC mass from global natural (fire) and regional anthropogenic (biofuel and fossil fuel) sources.
- Table S3. Observational data for Alert.
- Table S4. Observational data for Abisko.
- Table S5. Observational data for Barrow.
- Table S6. Observational data for Tiksi.
- Table S7. Observational data for Zeppelin.
- Table S8. Simulated fraction of BC mass (nonweighted) from global natural (fire) and regional anthropogenic (biofuel and fossil fuel) sources.
- References (*47*–*49*)

Download PDF

**Files in this Data Supplement:**

- Adobe PDF - aau8052\_SM.pdf
