# Supplementary material for: Source apportionment of circum-Arctic atmospheric black carbon from isotopes and modeling
Source: Sci Adv. 2019 Feb 13;5(2):eaau8052. doi: 10.1126/sciadv.aau8052 (PMC6374108; doi:10.1126/sciadv.aau8052)
Supplement: Download PDF [file aau8052_SM.pdf]

Science Advances
AAAS

advances.sciencemag.org/cgi/content/full/5/2/eaau8052/DC1

Supplementary Materials for

Source apportionment of circum-Arctic atmospheric black carbon from isotopes and modeling

P. Winiger*, T. E. Barrett, R. J. Sheesley, L. Huang, S. Sharma, L. A. Barrie, K. E. Yttri, N. Evangeliou, S. Eckhardt, A. Stohl, Z. Klimont, C. Heyes, I. P. Semiletov, O. V. Dudarev, A. Charkin, N. Shakhova, H. Holmstrand, A. Andersson, Ö. Gustafsson*

*Corresponding author. Email: p.winiger@vu.nl (P.W.); orjan.gustafsson@aces.su.se (Ö.G.)

Published 13 February 2019, *Sci. Adv.* **5**, eaau8052 (2019)
DOI: 10.1126/sciadv.aau8052

This PDF file includes:

Text S1. Gas-flaring uncertainties.
Fig. S1. Continental borders considered for the geographical sources in the FEG model.
Table S1. Seasonal observational data for the circum-Arctic.
Table S2. Simulated fraction of BC mass from global natural (fire) and regional anthropogenic (biofuel and fossil fuel) sources.
Table S3. Observational data for Alert.
Table S4. Observational data for Abisko.
Table S5. Observational data for Barrow.
Table S6. Observational data for Tiksi.
Table S7. Observational data for Zeppelin.
Table S8. Simulated fraction of BC mass (nonweighted) from global natural (fire) and regional anthropogenic (biofuel and fossil fuel) sources.
References (*47–49*)

Supplementary Materials

Text S1. Gas-flaring uncertainties.

The flaring conundrum has several layers, almost like the famous Matryoshka doll. The two main reasons on the outer layer could be, that flaring is either overestimated in the emission inventories (and other sources under estimated) or that our observations cannot quantify the flaring contribution, or both. To unravel the issue with observations, the next doll must be opened. Since all stations considered are remote from the major flaring fields in Russia, no pure flaring plumes were sampled, but only flaring emissions which were well mixed with other BC emissions, such as coal, which would shift the expected signature (from a more depleted, pure flaring $\delta^{13}C$ signature) to a more positive value. Long sampling times mix the signatures further, as can be seen for Barrow (Fig. 3). The samples collected at Barrow with a relatively short sampling time in winter/spring have a purer (narrower) source signature, closer to the *Regular* fossil fuels endmember, whereas samples with a prolonged sampling time are located relatively further away from the endmembers, i.e., in the 'mixed' zone where the bulk of all samples lie ($\delta^{13}C$ of –26.8‰, $\Delta^{14}C$ of –530‰). This leads us to the last layer and also a core issue in observations of gas flaring emissions. The isotopic endmember is relatively uncertain, and based on only one study (*47*). Shifts in the composition of the flared gas could mean a substantial change in isotopic ratio, as the kinetic isotopic effect during combustion affects small hydrocarbons (e.g., methane) more than larger molecules. There is thus a real lack of compound specific isotope analysis of pure gas flaring BC emissions. Coming back to the initial layer and the emission inventory uncertainties, we need to consider two main factors for uncertainty. This concerns the activity, that is the amount of gas flared, and the emission factor, which is the amount of BC emitted per unit gas, which also depends on the composition of flared gas, and the flare technology and its efficiency. A reason for the potential overestimation of gas flaring in previous studies could be the use of emission inventories from the year 2010 (as used in our model) or prior, while our observations were conducted during later years, when Russian flaring emissions had already started to decline (*48*), although the real extent of this decline in activity is uncertain. The emission factor uncertainty remains, although there are finally some measurements available (*49*) which point to the fact that emission factors can be relatively high, especially in mixtures that are rich in higher hydrocarbons (as found in Russia) and if flares are poorly operated, leading to worse local emissions than previously expected (*33*).

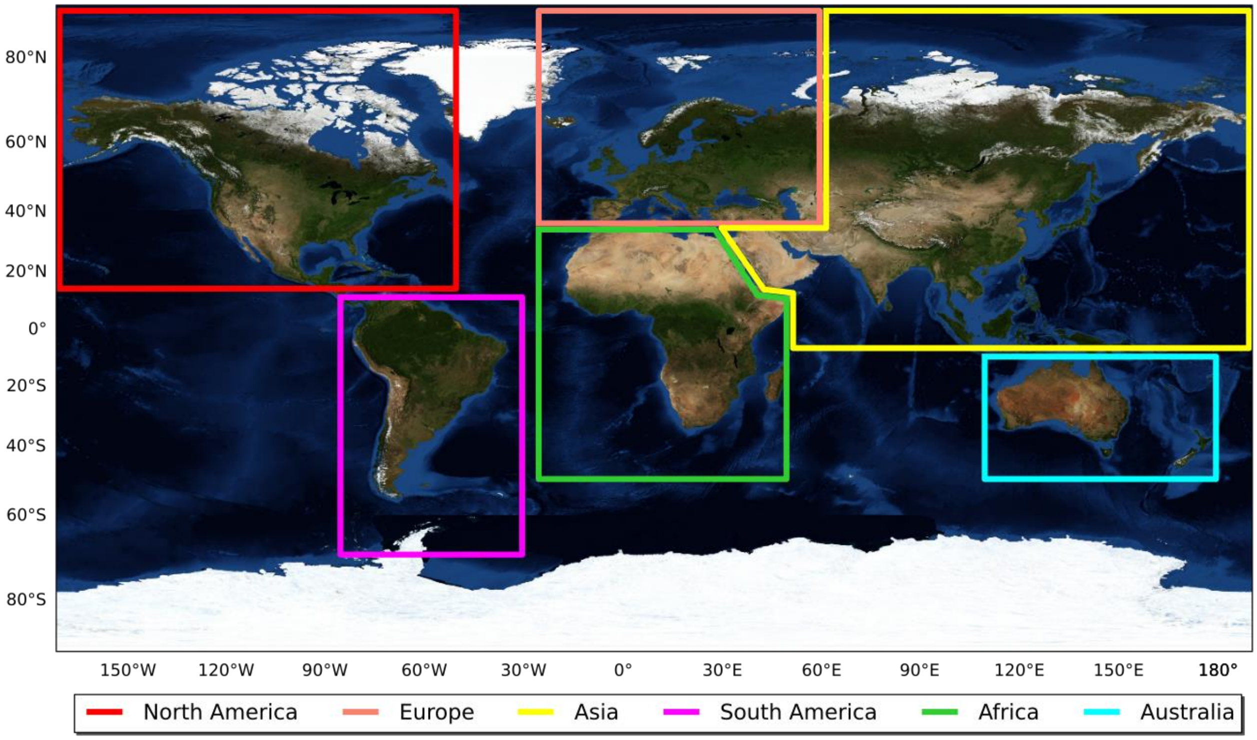

Fig. S1. Continental borders considered for the geographical sources in the FEG model.

Table S1. Seasonal observational data for the circum-Arctic. Winter (DJF), spring (MAM), summer (JJA) and autumn (SON). See Supplementary Tables 3 to 7 for single samples/composites.

Station	Season	Period DD.MM.YY - DD.MM.YY	Sample size #	Time [d]	Observation EC [ng C m^{-3}]			f_{bb} [-]			$\delta^{13}C$ [‰]		
Alert	DJF	05.11.14 - 18.03.15	2	133	60	±	17	0.399	±	0.046		-	
Alert	MAM	05.03.14 - 07.05.14	2	56	53	±	16	0.393	±	0.085	-27.1	±	0.1
Alert	JJA	07.05.14 - 30.07.14	1	84	12	±	0.6	0.392	±	0.053		-	
Alert	SON	30.07.14 - 05.11.14	1	98	8.2	±	0.4	0.374	±	0.054		-	
Abisko	DJF	20.12.11 - 22.02.12; 06.12.12 - 19.12.12	4	76	73	±	52	0.369	±	0.077	-25.8	±	1.8
Abisko	MAM	22.02.12 - 01.06.12	2	99	18	±	6.3	0.306	±	0.084	-27.4	±	0.0
Abisko	JJA	01.06.12 - 28.08.12	1	88	19	±	3.8	0.679	±	0.003	-26.0	±	0.2
Abisko	SON	28.08.12 - 06.12.12	2	100	14	±	6.8	0.415	±	0.127	-27.0	±	0.2
Barrow	DJF	26.11.12 - 01.03.13	6	58	40	±	8.7	0.105	±	0.090	-25.8	±	1.6
Barrow	MAM	01.03.13 - 04.06.13	6	70	16	±	24	0.260	±	0.153	-25.8	±	1.9
Barrow	JJA	16.07.12 - 21.09.12	2	46	13	±	0.4	0.328	±	0.263	-26.3	±	0.1
Barrow	SON	21.09.12 - 26.11.12	2	51	13	±	19	0.121	±	0.043		-	
Tiksi	DJF	02.01.14 - 07.03.14	3	63	44	±	3.2	0.323	±	0.030	-27.6	±	0.7
Tiksi	MAM	27.02.13 - 23.05.13	4	85	83	±	16	0.166	±	0.050	-29.1	±	0.3
Tiksi	JJA	23.05.13 - 25.07.13	1	63	41	±	3.3	0.362	±	0.038	-28.1	±	0.2
Tiksi	SON	25.07.13 - 02.01.14	2	162	13	±	7.9	0.604	±	0.003	-27.9	±	0.2
Zeppelin	DJF	15.11.12 - 23.02.13	2	100	19	±	0.5	0.359	±	0.016		-	
Zeppelin	MAM	23.02.13 - 19.04.13	4	52	27	±	10	0.392	±	0.026		-	
Zeppelin	JJA	19.04.13 - 12.07.13	1	84	4.2	±	0.2	0.616	±	0.052		-	
Zeppelin	SON	12.07.13 - 22.11.13	1	134	2.9	±	0.1	0.582	±	0.052		-	
Arctic	DJF	all DJF above	18		105	±	63	0.254	±	0.161		-	
Arctic	MAM	all MAM above	18		68	±	50	0.300	±	0.130		-	
Arctic	JJA	all JJA above	6		23	±	13	0.424	±	0.187		-	
Arctic	SON	all SON above	8		17	±	12	0.307	±	0.200		-	

Table S2. Simulated fraction of BC mass from global natural (fire) and regional anthropogenic (biofuel and fossil fuel) sources. Geographical sources from the FEG model. Average mass fractions and standard deviations are time-weighted. Model data for the summer 2013 is only partially complete due to ECMWF's increase of vertical model resolution (see Supplementary Table 8).

	Selected (annual) period		Time-weighted mass fraction of sources [%]											
Site	(DD.MM.YY)	Days	America			Asia			Europe			Fire		
Alert	05.03.14 - 18.03.15	371	2	±	1	50	±	16	27	±	10	21	±	25
Abisko	20.12.11 - 19.12.12	363	2	±	2	11	±	7	84	±	6	4	±	7
Barrow	16.07.12 - 04.06.13, not-continuous	224	17	±	11	43	±	21	23	±	13	17	±	27
Tiksi	27.02.13 - 07.03.14	310	0	±	3	41	±	25	12	±	9	47	±	30
Zeppelin	15.11.12 - 22.11.13	152	2	±	3	41	±	12	57	±	14	0	±	1
Arctic	All data		3	±	10	33	±	25	42	±	27	22	±	24
Arctic	All selected (annual)		3	±	11	36	±	25	38	±	25	23	±	24
Arctic	All selected (annual) w/o Barrow		1	±	2	35	±	27	40	±	27	24	±	21

Table S3. Observational data for Alert.

Start	Time	EC			f_{bb}			$\delta^{13}C$	
DD.MM.YY	[d]	[ng C m^{-3}]			[-]			[‰]	
12.02.14	20.7	53.6	±	2.7	0.422	±	0.052	-25.9	± 0.2
05.03.14	21.1	66.6	±	3.3	0.331	±	0.053	-27.1	± 0.2
26.03.14	34.8	44.0	±	2.2	0.451	±	0.053	-27.1	± 0.2
07.05.14	83.9	12.4	±	0.6	0.392	±	0.053	-	
30.07.14	98.1	8.2	±	0.4	0.374	±	0.054	-	
05.11.14	56.1	44.7	±	2.2	0.401	±	0.053	-	
31.12.14	41.8	66.6	±	3.3	0.444	±	0.053	-27.7	± 0.2
11.02.15	34.9	76.5	±	3.8	0.352	±	0.052	-29.0	± 0.2
18.03.15	35.0	46.5	±	2.3	0.397	±	0.052	-26.8	± 0.2

Table S4. Observational data for Abisko.

Start	Time	EC			f_{bb}			$\delta^{13}C$	
DD.MM.YY	[d]	[ng C m^{-3}]			[-]			[‰]	
29.09.11	28.0	22	±	17	0.343	±	0.002	-26.0	± 0.2
27.10.11	12.8	23	±	1.7	0.306	±	0.002	-26.6	± 0.2
08.11.11	21.0	24	±	1.5	0.407	±	0.001	-26.7	± 0.2
29.11.11	21.0	19	±	1.3	0.295	±	0.002	-25.1	± 0.2
20.12.11	21.0	24	±	1.6	0.171	±	0.004	-27.9	± 0.2
11.01.12	21.0	118	±	6.3	0.415	±	0.001	-24.1	± 0.2
01.02.12	20.8	45	±	2.6	0.374	±	0.002	-27.1	± 0.2
22.02.12	41.0	23	±	4.5	0.250	±	0.003	-27.4	± 0.2
04.04.12	58.0	14	±	1.4	0.370	±	0.002	-27.4	± 0.2
01.06.12	88.0	19	±	3.8	0.679	±	0.003	-26.0	± 0.2
28.08.12	53.2	10	±	10	0.510	±	0.002	-26.9	± 0.2
20.10.12	46.8	20	±	5.0	0.330	±	0.002	-27.2	± 0.2
06.12.12	13.0	131	±	5.5	0.360	±	0.002	-27.0	± 0.2
19.12.12	14.0	117	±	4.9	0.345	±	0.002	-27.8	± 0.2
02.01.13	28.0	32	±	22	0.235	±	0.003	-	
30.01.13	30.0	45	±	4.6	0.438	±	0.002	-26.6	± 0.2
01.03.13	26.0	39	±	15	0.467	±	0.002	-26.7	± 0.2

Table S5. Observational data for Barrow. The symbols indicate samples, which were combined to one composite before the isotope analysis. The six samples from 28.12.2012 to 20.03.2013 have been analysed differently, as published in Barret et al. (2015).

Start	Time	EC			f_{bb}			$\delta^{13}C$		
DD.MM.YY	[d]	[ng C m^{-3}]			[-]			[‰]		
16.07.12	6.8	12.5	±	0.2	0.154	±	0.053	-26.3	± 0.2	*
23.07.12	11.0	13.0	±	4.8	0.527	±	0.054	-26.4	± 0.2	†
03.08.12	9.5	12.5	±	0.2	0.154	±	0.053	-26.3	± 0.2	*
12.08.12	18.5	13.0	±	4.8	0.527	±	0.054	-26.4	± 0.2	†
21.09.12	14.0	6.4	±	8.3	0.081	±	0.054	-		‡
12.10.12	13.9	6.4	±	8.3	0.081	±	0.054	-		‡
03.11.12	23.1	38.3	±	6.1	0.144	±	0.053	-27.2	± 0.2	
26.11.12	24.8	34.0	±	11.7	0.080	±	0.053	-26.8	± 0.2	
28.12.12	8.1	40.1	±	8.1	0.336	±	0.092	-24.0	± 0.3	
18.01.13	6.9	77.0	±	10.7	0.325	±	0.092	-25.0	± 0.4	
01.02.13	6.9	67.3	±	13.3	0.289	±	0.092	-26.8	± 0.5	
08.02.13	7.0	97.1	±	11.0	0.315	±	0.092	-26.8	± 0.6	
25.02.13	4.1	35.8	±	14.1	0.505	±	0.092	-21.8	± 0.7	
01.03.13	9.9	41.5	±	6.5	0.513	±	0.092	-22.8	± 0.8	
20.03.13	9.0	36.7	±	2.8	0.104	±	0.053	-25.7	± 0.2	**
04.04.13	7.8	36.7	±	2.8	0.104	±	0.053	-25.7	± 0.2	**
12.04.13	14.0	50.7	±	5.4	0.105	±	0.053	-26.2	± 0.2	
03.05.13	7.1	41.3	±	7.0	0.047	±	0.052	-26.8	± 0.2	
10.05.13	13.9	22.4	±	2.3	0.104	±	0.054	-26.9	± 0.2	
27.05.13	8.1	2.7	±	7.5	0.202	±	0.053	-27.7	± 0.2	

Table S6. Observational data for Tiksi.

Start	Time	EC			f_{bb}			$\delta^{13}C$	
DD.MM.YY	[d]	[ng C m^{-3}]			[-]			[‰]	
16.04.12	66.9	67.7	±	5.5	0.408	±	0.026	-28.2	± 0.2
21.06.12	63.0	37.8	±	4.3	0.731	±	0.049	-25.8	± 0.2
24.08.12	62.0	19.8	±	3.1	0.619	±	0.046	-27.5	± 0.2
25.10.12	63.0	21.2	±	3.3	0.253	±	0.018	-30.1	± 0.2
27.12.12	17.9	73.5	±	6.4	0.194	±	0.029	-29.2	± 0.2
06.02.13	21.0	302.1	±	16.2	0.080	±	0.023	-30.7	± 0.2
27.02.13	21.0	82.5	±	5.3	0.119	±	0.028	-29.2	± 0.2
20.03.13	21.0	85.8	±	5.5	0.187	±	0.031	-29.0	± 0.2
10.04.13	22.0	103.2	±	6.5	0.141	±	0.031	-29.4	± 0.2
02.05.13	21.0	63.0	±	4.4	0.239	±	0.034	-28.6	± 0.2
23.05.13	63.0	40.8	±	3.3	0.362	±	0.038	-28.1	± 0.2
25.07.13	84.0	8.0	±	1.5	0.600	±	0.034	-27.6	± 0.2
17.10.13	78.0	19.2	±	2.1	0.605	±	0.015	-28.0	± 0.2
02.01.14	20.1	41.0	±	3.1	0.359	±	0.027	-27.5	± 0.2
23.01.14	21.9	47.2	±	3.4	0.313	±	0.023	-28.2	± 0.2
14.02.14	21.0	43.0	±	3.2	0.300	±	0.027	-27.0	± 0.2
07.03.14	21.0	37.3	±	3.0	0.263	±	0.028	-26.9	± 0.2

Table S7. Observational data for Zeppelin.

Start	Time	EC			f_{bb}			$\delta^{13}C$
DD.MM.YY	[d]	[ng C m^{-3}]			[-]			[‰]
15.11.12	70.9	19.7	±	1.0	0.350	±	0.053	-
25.01.13	29.0	19.0	±	0.9	0.373	±	0.052	-
23.02.13	13.0	32.9	±	1.6	0.372	±	0.052	-
11.03.13	14.0	13.8	±	0.7	0.423	±	0.052	-
25.03.13	10.9	34.3	±	1.7	0.372	±	0.052	-
05.04.13	14.0	29.5	±	1.5	0.414	±	0.052	-
19.04.13	84.1	4.2	±	0.2	0.616	±	0.052	-
12.07.13	134.1	2.9	±	0.1	0.582	±	0.052	-
22.11.13	14.0	20.6	±	1.0	0.466	±	0.052	-
06.12.13	13.9	14.4	±	0.7	0.358	±	0.055	-
23.12.13	7.0	36.7	±	1.8	0.337	±	0.052	-

Table S8. Simulated fraction of BC mass (nonweighted) from global natural (fire) and regional anthropogenic (biofuel and fossil fuel) sources. geographical sources from the FEG model. Model data for the summer 2013 is only partially complete due to ECMWF's increase of vertical model resolution.

Station	Season	Period DD.MM.YY	Time [d]	Fire [%]	Biofuel America [%]	Biofuel Europe [%]	Biofuel Asia [%]	Fossil fuel America [%]	Fossil fuel Europe [%]	Fossil fuel Asia [%]
Alert	DJF	05.11.14 - 18.03.15	133	1	0	6	4	1	28	58
Alert	MAM	05.03.14 - 07.05.14	56	6	1	7	5	2	27	53
Alert	JJA	07.05.14 - 30.07.14	84	37	1	4	8	3	13	35
Alert	SON	30.07.14 - 05.11.14	98	66	1	2	1	2	10	18
Abisko	DJF	20.12.11 - 22.02.12 and 06.12.12 - 19.12.12	76	0	0	23	1	1	28	8
Abisko	MAM	22.02.12 - 01.06.12	99	3	1	25	1	3	57	10
Abisko	JJA	01.06.12 - 28.08.12	88	22	1	29	0	3	44	1
Abisko	SON	28.08.12 - 06.12.12	100	3	0	25	0	1	65	5
Barrow	DJF	26.11.12 - 01.03.13	58	0	3	5	3	7	30	52
Barrow	MAM	01.03.13 - 04.06.13	70	5	6	3	5	13	23	45
Barrow	JJA	16.07.12 - 21.09.12	46	75	5	1	1	9	4	5
Barrow	SON	21.09.12 - 26.11.12	51	5	9	4	5	19	19	38
Tiksi	DJF	02.01.14 - 07.03.14	63	0	0	2	2	0	16	80
Tiksi	MAM	27.02.13 - 23.05.13	85	7	0	2	4	1	27	59
Tiksi	JJA	23.05.13 - 25.07.13				No data				
Tiksi	SON	25.07.13 - 02.01.14	162	69	0	1	1	0	6	22
Zeppelin	DJF	15.11.12 - 23.02.13	100	0	0	10	1	1	51	37
Zeppelin	MAM	23.02.13 - 19.04.13	52	1	1	4	3	3	34	54
Zeppelin	JJA	19.04.13 - 12.07.13				No data				
Zeppelin	SON	12.07.13 - 22.11.13				No data				
